# Lurasidone augmentation to clozapine in treatment resistant schizophrenia: A pilot study

**DOI:** 10.1192/j.eurpsy.2023.684

**Published:** 2023-07-19

**Authors:** V. Arienti, S. C. Civardi, F. Besana, F. Mazzoni, G. Carnevale Miacca, N. Brondino, P. Politi, M. Olivola

**Affiliations:** ^1^Dipartimento di Scienze del Sistema Nervoso e del Comportamento, Università degli studi di Pavia; ^2^Dipartimento di Salute Mentale e Dipendenze, ASST Pavia, Pavia, Italy

## Abstract

**Introduction:**

Treatment resistant schizophrenia still represents a major clinical and pharmacological challenge.30% of patients diagnosed with schizophrenia is characterised by a poor response to at least two different antipsychotics administered for a proper period of time and at adequate doses. Clozapine still represents the gold standard for treatment resistant patients. Unfortunately, a significant percentage of these are only partial responders. Augmentation strategies must be set up and atypical antipsychotic drugs are used in clinical practice. Promising findings have been observed in patients treated with Lurasidone as an add-on therapy with Clozapine. This novel second-generation antipsychotic has a unique receptor profile, showing 5-HT1a partial agonism and 5HT7 antagonism. These properties could also explain its procognitive effect, as several preclinical studies in literature have demonstrated.

**Objectives:**

The aim of our study is to highlight the advantages of add on therapy with Lurasidone compared with treatment as usual (i.e. Clozapine + another atypical antipsychotic) in treatment resistant schizophrenia patients.

**Methods:**

We conducted an observational study in a sample of 20 patients diagnosed with treatment resistant schizophrenia, based on DSM-5 diagnostic criteria and psychopharmacologic history. Treatment choices were taken independently by clinicians in charge of each patient. 10 subjects underwent Lurasidone augmentation of Clozapine, whereas the remaining 10 subjects were treated as usual with Clozapine and another atypical antipsychotic. PANSS and BPRS scales to assess general psychopathology and UKU side effects scale were administered both at baseline and at follow-up (T1= 1 month; T2=6 months).

**Results:**

All patients treated with Lurasidone augmentation strategy achieved a significant reduction of both positive and negative symptoms, with no significant adverse effects to be reported. In particular, Lurasidone showed no impact on metabolic parameters nor on ECG features, namely the QTc interval. The psychopathological improvement appeared higher in patients who received Lurasidone than in those treated as usual. This was particularly evident in cognitive domains.

**Conclusions:**

Our observation suggests that augmentation strategy with Lurasidone to Clozapine can lead to clinically significant improvements in psychopathology when compared to Clozapine combined with another atypical antipsychotic, with a good tolerability profile. In future we will increase the number of our sample and the duration of follow-up time. In order to have more relevant statistical results, further research on this topic is needed.

**Disclosure of Interest:**

None Declared

